# High frequency of social polygyny reveals little costs for females in a songbird

**DOI:** 10.1038/s41598-021-04423-0

**Published:** 2022-01-07

**Authors:** Simone Santoro, Pilar Fernández-Díaz, David Canal, Carlos Camacho, László Z. Garamszegi, Jesús Martínez-Padilla, Jaime Potti

**Affiliations:** 1 Department of Integrated Sciences, Faculty of Experimental Sciences, University of Huelva, 21007 Huelva, Spain; 2 Department of Molecular Biology and Biochemical Engineering, University Pablo de Olavide, Seville, Spain; 3 Department of Evolutionary Ecology, Estación Biológica de Doñana (CSIC), Seville, Spain; 4 Institute of Ecology and Botany, Centre for Ecological Research, Vácrátót, Alkotmány u. 2-4, Hungary; 5 Department of Biological Conservation and Ecosystem Restoration, Pyrenean Institute of Ecology (CSIC), Jaca, Spain; 6 MTA-ELTE, Theoretical Biology and Evolutionary Ecology Research Group, Department of Plant Systematics, Ecology and Theoretical Biology, Eötvös Loránd University, Budapest, Hungary

**Keywords:** Ecological modelling, Evolutionary ecology, Population dynamics

## Abstract

Mating system theory predicts that social polygyny—when one male forms pair bonds with two females—may evolve by female choice in species with biparental care. Females will accept a polygynous male if the benefit of mating with a male providing high-quality genes or rearing resources outweighs the cost of sharing mate assistance in parental care. Based on this rationale, we hypothesise that the population frequency of social polygyny (FSP) varies due to changes in mate sharing costs caused by changing environmental conditions. We predicted that: (1) polygamous females (i.e. mated with a polygynous male) pay a survival cost compared to monogamous females; (2) FSP would be higher in years with better rearing conditions and (3) the difference in survival rates between monogamous and polygamous females would be small following years with higher FSP. We tested these predictions using regression and multistate analyses of capture-recapture data of pied flycatchers, *Ficedula hypoleuca*, in central Spain collected over 26 years (1990–2016). Monogamous females had a higher mean survival rate than polygamous females (prediction 1), but there was no difference in survival between polygynous and monogamous males. In addition, FSP was positively associated with annual reproductive success (a proxy of the quality of rearing conditions—prediction 2). Finally, following years with high FSP, the survival of polygamous females was similar to that of monogamous females (prediction 3), while the chance of breeding in a polygamous state for 2 years in a row increased for both males and females. Our findings suggest that fluctuating environmental conditions may be a necessary but neglected aspect of understanding social polygyny mechanisms.

## Introduction

Males often invest less in parental care than females during reproduction, allowing them to spend more time and energy searching for additional mating opportunities^1^. Polygyny—when males pair with multiple females and females with one male—is the most common form of polygamy. This mating strategy occurs in various taxa and is ubiquitous in some groups (e.g., birds and mammals^2,3^). Social polygyny (and extra-pair fertilisations; not discussed in this study) should be beneficial for males because, by mating with several females, they increase their reproductive fitness^4,5^. In contrast, the benefits of social polygyny for females are less clear as sharing critical resources, such as nest sites, food and male parental care, with another female should result in fitness costs^1,6,7^. Broad theoretical and empirical research has examined the evolutionary mechanisms that generate and maintain social polygyny^6–10^. The theoretical side of this topic has been dominated by the polygyny threshold model (PTM—^8^), the sexy son hypothesis (SSH—^9^), and the deception hypothesis^11^. According to the PTM and the SSH^8,9^, females will choose an already-mated male when the quality of the “breeding situation”^12^ he provides overcomes the cost of mate sharing so that the female breeding prospects are greater than those accrued from mating with an unpaired male. The “*breeding situation*” term indicates the combined quality of the male (genetic and parental care) and the environmental conditions at the nest site or territory^12^. According to the deception hypothesis, mated males would hide their mating status and deceive females into polygyny^11^. Despite the large body of previous work, empirical results are inconsistent, and why females would mate with already-mated males remains hotly debated^13,14^.

Trade-offs between investment in current and future reproduction often result in survival costs (i.e., cost of reproduction)^15^. In biparental avian species, females mated to polygynous males (hereafter “polygamous females”) may increase their investment in parental care to compensate for the reduced assistance of their mates^16–18^. Polygamous female survival, lifetime reproductive success and mating status in a passerine bird are thus predicted to have lower survival than monogamous females because of the trade-off between their current reproductive effort and future survival. In contrast, it is widely assumed that males pay a negligible survival cost by mating with multiple females, although this has rarely been tested^19–21^. The empirical evidence for polygyny's survival costs is mixed, indicating both costs^19,20,22^ and benefits^23^.

**Figure 1:**
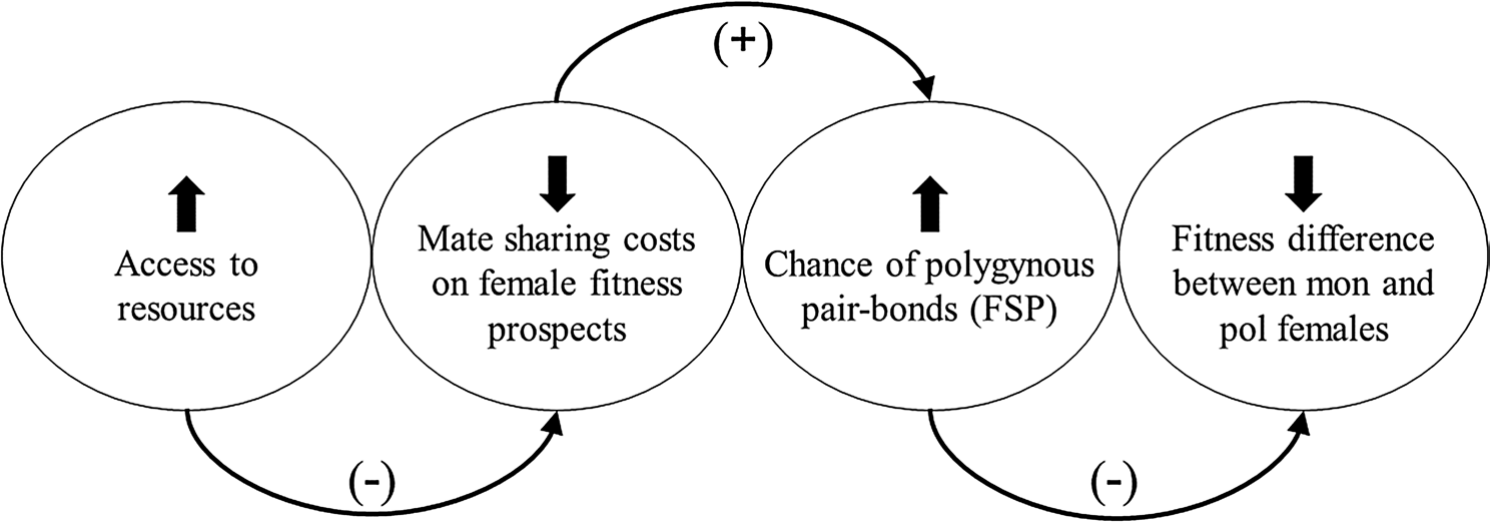
A schematic diagram of the EPT hypothesis. When there is easy access to the resources for rearing offspring, the expected costs of mate sharing on polygamous females’ fitness are negligible. Females are more willing to accept an already-mated male in these circumstances, increasing the frequency of social polygyny (FSP) in the population. The fitness difference, measured in this study as survival after the breeding season, between monogamous (mon) and polygamous (pol) females narrows or even disappears.

The frequency of social polygyny (FSP, i.e., the proportion of polygynous breeding males in a population) varies within and among species (reviewed in passerines^2^) and populations (^24^, our study population). Three hypotheses might explain this variation. First^1^, the operational sex ratio (the proportion of reproductively active males to females) may increase male intra-sexual competition such that some high-quality males monopolise more than one female and others do not breed, resulting in a higher FSP. Second, the deception hypothesis proposes that high breeding density and resource competition may reduce the ability of an already-mated male to conceal his mating status to prospecting females^11^, resulting in a lower FSP. Third, FSP may fluctuate if females’ willingness to accept an already-paired male varies from year to year depending on the changes in the costs of mate sharing due to variation in environmental conditions. Since the polygyny threshold, i.e. the difference in quality of breeding situations required for a polygynous mating to be advantageous to a female, changes if the costs of mate sharing for females change, we define the *fluctuating polygyny threshold* (FPT) *hypothesis* as follows: when favourable environmental conditions improve the breeding situation for females, irrespective of their mating status (monogamous or polygamous), the females’ mate sharing costs will be predictably low, making them more willing to accept an already-mated male because of better fitness prospects (Fig. 1).

The pied flycatcher, *Ficedula hypoleuca*, a facultatively polygynous species with large interannual variation in FSP, was the model organism of the present study. The pied flycatcher is a small insectivorous, migratory, hole-nesting passerine with biparental care. Upon arrival to the breeding areas, males search for a nest site, compete for its possession, defend it from intruders, attract and mate with a female, and provide parental care (e.g. feeding and defence^16^) to the offspring. The species is typically monogamous but, after mating with a female (which in this case becomes the primary female), some males (3–25%)^16^ occupy another cavity and attract a second female (namely, the secondary female), becoming socially polygynous. Polygynous males often provide little, or even no, parental care to their secondary broods, reducing reproductive success in those nests^11^. Some studies have shown that males can improve their reproductive success from mating polygynously^25,26^, but environmental conditions may modulate this effect (e.g., food availability^27^).

This study examines whether the current mating status (monogamous or polygamous) of males and females affects their survival and future mating status and how these processes are associated with the frequency of social polygyny in the population. For this purpose, we performed regression and a multistate capture-recapture analyses using 26 years of breeding data from a Spanish population of pied flycatchers by treating mating status as a sex-specific, dynamic individual trait (i.e. potentially changing between years). More specifically, we tested three predictions of the FPT hypothesis. First, because male attendance at secondary nests is typically lower in facultatively polygynous species, we predicted higher survival probabilities for the monogamous females, followed by primary and secondary females. In our study population, bigamous males often only assist the secondary female in feeding the nestlings after the primary brood has fledged^24,28^. Also, although the relevance of post-fledging parental care is unknown, primary females may suffer from a lack of male attendance after the primary brood has fledged. By contrast, we did not predict differences in survival between monogamous and polygynous males because male parental care in this and other bird species is typically reduced or absent in secondary broods^16–18^. Second, we predicted that the FSP would rise in years with better fledging success (a proxy of the quality of rearing conditions) because the polygyny threshold would fall when the costs of mate sharing are low due to better rearing conditions. Third, we predicted that the survival of polygamous females would approach that of monogamous females following seasons with high FSP relative to years with low FSP. This is a critical prediction, not compatible with other hypotheses about the drivers of FSP variation, like OSR^1^ or breeding density^11^, as they do not predict any association between the FSP and the differential survival of females in relation to their mating status.

## Results

### Females

**Figure 2:**
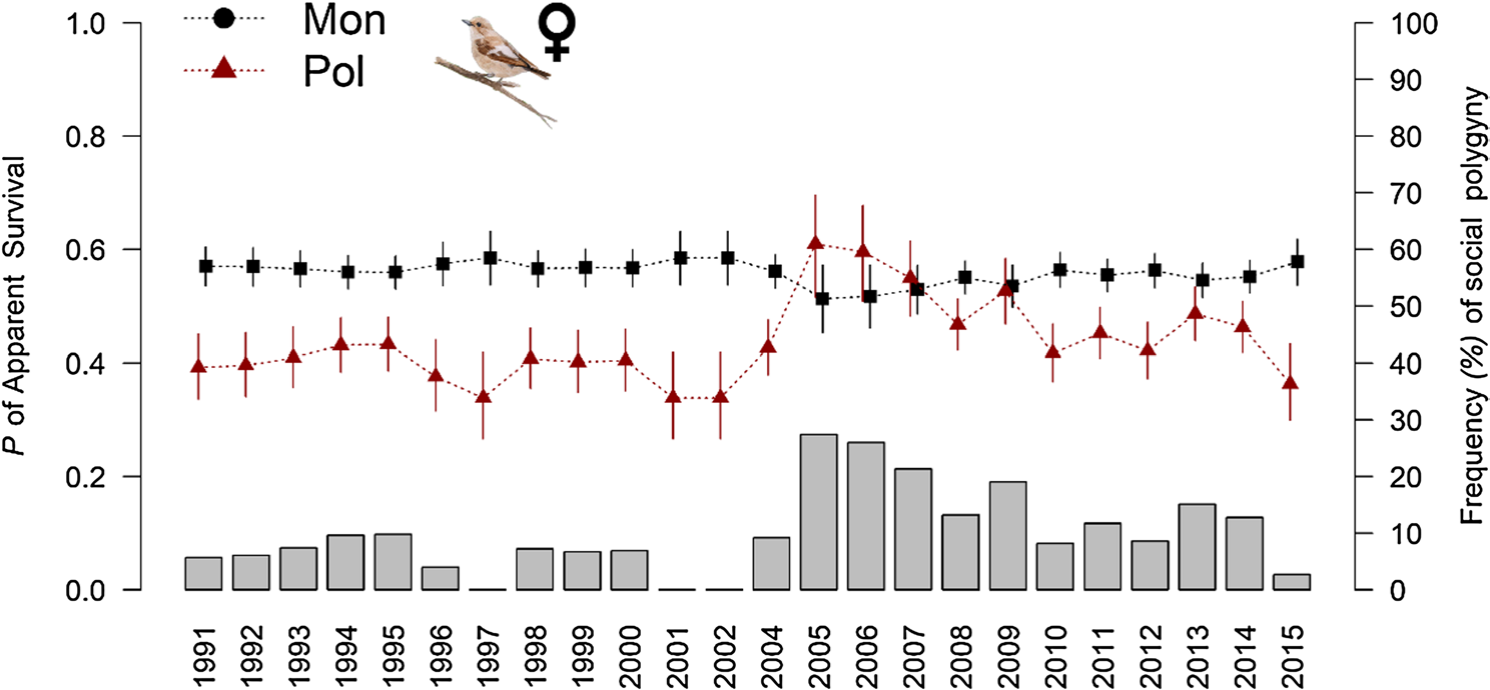
The relationship between local survival probabilities, frequency of social polygyny (FSP), and mating status. 95%CI estimates of female annual local survival according to their mating status (monogamous vs polygamous) and FSP (grey bars) in the previous breeding season. *Mon* = monogamous, *Pol* = polygamous (primary or secondary). Only the estimates of > 1-yo females (the effect of age was additive, see Table 1) are shown.

The local survival probability of polygamous females was, on average, higher than that of monogamous females (FPT hypothesis: prediction 1) and also for 1yo females compared to older females (all the estimates are from model F6: P_mon 1-yo_ = 0.63, 95%CI = 0.58–0.68, P_pol 1-yo_ = 0.55, 95%CI = 0.49–0.6, P_mon >1-yo_ = 0.55, 95%CI = 0.52–0.58, P_pol >1-yo_ = 0.47, 95%CI = 0.42–0.51). Local survival of primary and secondary females did not differ significantly (Table 1). When the FSP in the previous breeding season was high, the polygamous female’ local survival approached that of monogamous females (FPT hypothesis: prediction 3—Fig. 2, Table 2).

**Table 1:** Model selection for mating status change and local survival probabilities.

No.	Model	np	Dev	AIC_c_	ΔAIC_c_	No.	Model	np	Dev	AIC_c_	ΔAIC_c_
**Females—mating status change**						**Males—mating status change**					
F1	*mon.pol1*	138	8754.89	9043.89	0	M1	*ms*	107	5582.81	5806.1	0
F2	*ms*	141	8749.72	9045.3	1.41	M2	*age*+ *ms*	108	5580.87	5806.34	0.23
F3	*age x mon.pol1*	139	8754.11	9045.3	1.42	M3	*age x ms*	109	5580.68	5808.32	2.22
F4	*mon.pol2*	139	8755.18	9046.37	2.49	M4	*age*	107	5649.63	5872.92	66.82
F5	*age x ms*	147	8742.87	9051.65	7.77	M5	*constant*	106	5652.3	5873.42	67.31
**Females—local survival**						**Males—local survival**					
F6	*age* + *ms (M,P* = *S)*	65	8861.19	8994.02	0	M6	*constant*	57	5641.33	5757.93	0
F7	*age* + *ms (M* = *P,S)*	65	8863.12	8995.96	1.93	M7	*age*	58	5640.23	5758.92	0.99
F9	*age* + *ms*	66	8861.14	8996.07	2.04	M8	*ms*	58	5641.22	5759.92	1.98
F10	*age*	64	8870	9000.75	6.72	M9	*t*	81	5600.59	5767.87	9.94
F11	*ms (M,P* = *S)*	64	8871.82	9002.57	8.55	M10	*ms*+ *t*	82	5600.58	5770	12.07
F12	*ms*	65	8871.8	9004.64	10.62	M11	*ms x t*	82	5583.76	5802.7	44.77
F13	*ms (M* = *P,S)*	64	8874.11	9004.86	10.83	M12	*age*+ *ms x t*	107	5582.81	5806.1	48.17
F14	*constant*	63	8879.08	9007.74	13.72						
F15	*age* + *ms* + *t*	90	8834.59	9020.05	26.03						
F1	*age* + *ms x t*	138	8754.89	9043.89	49.86						

For each model, the deviance, the number of estimable parameters (*np*), the Akaike Information Criterion corrected for small sample sizes (AIC_c_), and the difference in AIC_c_ between the current model and best model with the lowest AIC_c_ of the current parameter (ΔAIC_c_) are shown. For other parameters, see Table S1 for model selection.

Model notation: +, additive relationship, *x*, non-additive relationship, *ms*, different *P* of change from one mating status to another or a different survival *P* for each mating status, *mon.pol1*, is a set of three constrained probabilties: (i) same *P* of change from "mon to prim" and "mon to sec", (ii) same *P* of change from "prim to prim" and "sec to sec", and (iii) same *P* of change from "prim to sec" and "sec to prim", *mon.pol2*, like *mon.pol1* but different *P* of “prim to sec” and “sec to prim”, *ms (M,P* = *S)*, same survival *P* of prim and sec, but different from mon females, *ms (M* = *P,S)*, same survival *P* of mon and prim, but distinct from sec females, *t*, time; *constant*, no effect, *age*, two-classes age effect (1-yo; > 1-yo).

**Table 2:** Model selection to test the effect of frequency of social polygyny on the probabilities of mating status change and local survival probabilities of pied flycatcher females and males.

No.	Model	np	Dev	AIC_c_	ΔAIC_c_	No.	Model	np	Dev	AIC_c_	ΔAIC_c_
**Females—mating status change**						**Males—mating status change**					
F-FSP1	*pol.pol x FSP*	66	8854.67	8989.6	0	M-FSP1	*pol.pol x FSP*	58	5637.48	5756.18	0
F-FSP2	*prim.sec-sec.prim x FSP*	66	8858.42	8993.34	3.75	M6	*ms*	57	5641.33	5757.93	1.75
F6	*mon.pol*	65	8861.19	8994.02	4.43	M-FSP2	*mon.mon x FSP*	58	5640.75	5759.45	3.26
F-FSP3	*mon.pol x FSP*	66	8860.82	8995.75	6.15						
**Females—local survival**						**Males—local survival**					
F-FSP4	*mon x FSP pol x FSP*	67	8849.12	8986.13	0	M6	*constant*	57	5641.33	5757.93	0
F-FSP5	*pol x FSP*	66	8851.98	8986.91	0.77	M-FSP3	*mon x FSP*	59	5640.23	5761.02	3.09
F6	*ms (M,P* = *S)*	65	8861.19	8994.02	7.89	M-FSP4	*pol x FSP*	59	5641.12	5761.91	3.98
F-FSP6	*mon x FSP*	66	8859.61	8994.53	8.4	M-FSP5	*mon x FSP pol x FSP*	60	5640.22	5763.11	5.17

For each model, we report the deviance, the number of estimable parameters (*np*), the Akaike Information Criterion corrected for small sample sizes (AIC_c_) and the difference in AIC_c_ between the current model and best model with the lowest AIC_c_ of the current parameter (ΔAIC_c_).

Model notation: *pol.pol*, *P* of mating status change from “pol to pol” (females: primary or secondary, males: polygynous), *mon.mon*, *P* of “mon to mon”, *prim.sec-sec.prim*, same *P* of “prim to sec” and “sec to prim”, *mon.pol*, this is the null model without any effect of FSP on the *P* of mating status change (corresponding to *mon.pol1* in Table 1), *ms*, this is the null model without any effect of FSP on the *P* of survival (see *ms* in Table 1), *mon*, monogamous, *pol*, polygamous, *prim*, primary, *sec*, secondary, *x FSP*, that depends on the frequency of social polygyny, *constant*, no effect. Note that in *Females*—*Local survival*, although not explicitly stated, all the models include the additive effect of *age* (see Table 1).

**Figure 3:**
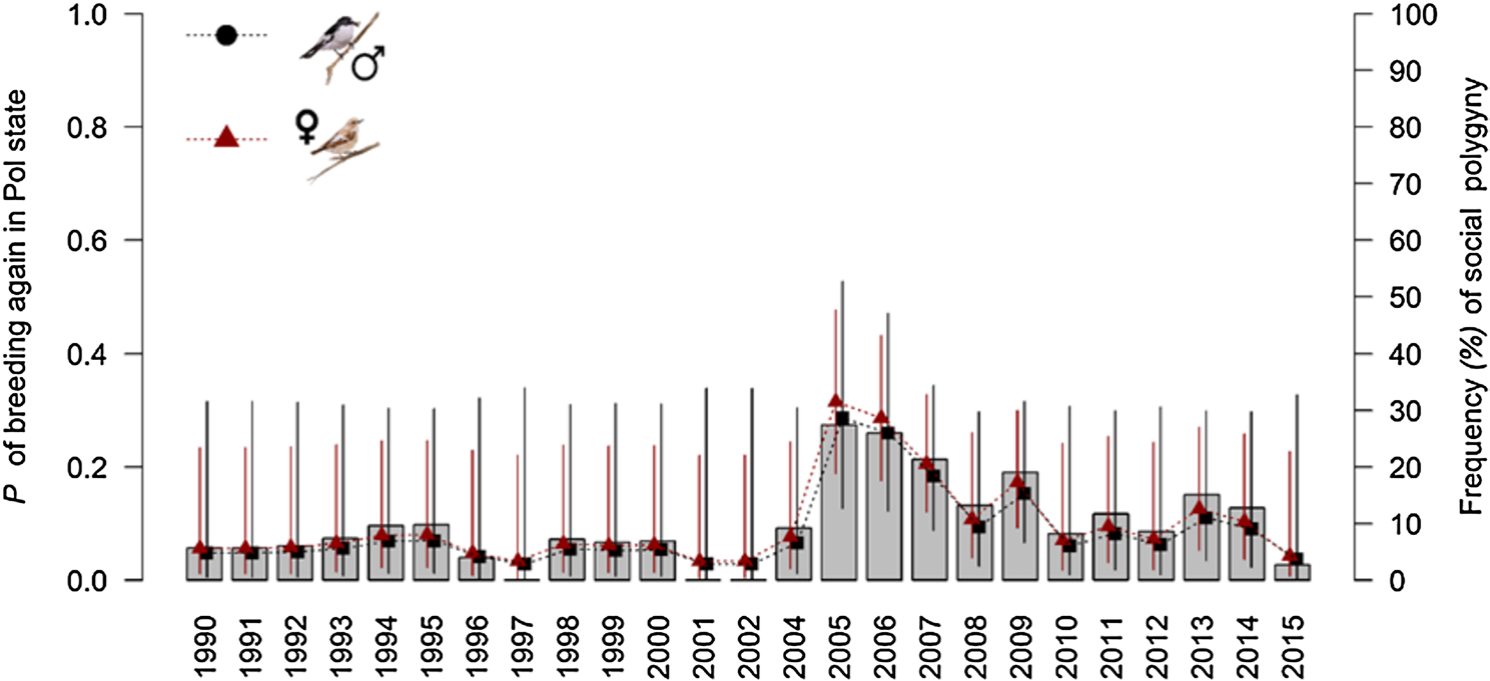
95%CI estimates of the probability of breeding two consecutive years in a polygamous state according to the frequency of social polygyny (grey bars) in the previous year for males and females separately.

In the next breeding season, a monogamous female had the same chance of becoming primary or secondary (Table 1, P_mon to prim_ = P_mon to sec_ = 0.15, 95%CI = 0.13–0.17). Also, a primary or secondary female had the same chance of becoming monogamous (P_prim to mon_ = P_sec to mon_ = 0.7, 95%CI = 0.63–0.75) and the same chance of changing from one polygamous state to another (P_prim to sec_ = P_sec to prim_ = 0.11, 95%CI 0.05–0.24). Following seasons of high FSP, the probability of breeding two consecutive years in a polygamous state (primary or secondary) increased (Fig. 3, Table 2).

Unsurprisingly, considering that monogamous pairs are much more common than polygamous ones (Appendix S1), the individual probability of being monogamous at the first known breeding event was considerably higher than that of being polygamous. More specifically, at their first breeding event, older females were more likely than younger females to be monogamous and less likely to be secondary (P_>1-yo mon_ = 0.82, 95% CI = 0.79–0.85, P_1-yo mon_ = 0.75, 95% CI = 0.72–0.79, P_>1-yo prim_ = 0.07, 95% CI = 0.04–0.11, P_1-yo prim_ = 0.07, 95% CI = 0.04–0.14, P_>1-yo sec_ = 0.11, 95% CI = 0.08–0.15 P_1-yo sec_ = 0.17, 95% CI = 0.13–0.23). For the complete list of parameters’ estimates (including all the complementary probabilities and the probabilities of recapture and mating status assignment) see Appendix S2.

### Males

We did not detect any effect of mating status, year, age, or FSP on the local survival of monogamous and polygynous males (Table 2; P_mon=pol_ = 0.54, 95%CI 0.52–0.56). Polygynous males were more likely to change their mating status to monogamous than to remain polygynous in consecutive breeding seasons (estimates from model M6 in Table 2: P_pol to mon_ = 0.85, 95%CI = 0.74–0.92, P_pol to pol_ = 0.15, 95%CI = 0.08–0.26). In contrast, monogamous males were more likely to maintain their monogamous mating status in subsequent years (all the estimates are from model M6: P_mon to mon_ = 0.79, 95%CI = 0.75–0.83) than to become polygynous (model M6: P_mon to pol_ = 0.21, 95%CI = 0.17–0.25). Similar to females, when the FSP was high in 1 year, a polygynous male was more likely to be polygynous the following year (Table 2, Fig. 3).

At the first known breeding event, a male was more likely to be monogamous than polygynous (P_1-yo mon_ = 0.99, 95%CI = 0.97–1.00, P_>1-yo mon_ = 0.96, 95%CI = 0.95–0.97). However, contrary to females, older (> 1-yo) males were more likely to be polygynous than monogamous (P_1-yo pol_ = 0.01, 95%CI = 0.00–0.03, P_>1-yo pol_ = 0.04, 95%CI = 0.03–0.05; see Appendix S2 for all the other estimates).

### FSP and fledging success of hatchlings

The FSP was positively associated to the yearly average proportion of hatchlings that fledged ((FPT hypothesis: prediction 3—*β*_FSP_ = 0.64, SE = 0.31, likelihood ratio test *P* = 0.04).

## Discussion

In this study, we proposed the *fluctuating polygyny threshold hypothesis* stating that year-to-year changing costs of mate sharing for females modify the polygyny threshold they must overcome before accepting a mated male. We found observational support to this hypothesis using a long-term study of the pied flycatcher to model mating status as a sex-specific dynamic individual trait. Monogamous females had a higher survival rate than polygamous (primary or secondary) females. In contrast, polygynous males did not pay any survival cost. The lower survival of polygamous females is probably due to increased breeding investment to compensate for polygynous males’ little assistance^16–18^. Also, we found a positive association between the frequency of social polygyny and the proportion of hatchlings that managed to fledge, a proxy of the quality of rearing conditions. Finally, following years with a high frequency of social polygyny in the population, the survival of polygamous females approached that of monogamous females, and the chances that a polygamous female or male had the same mating status in the subsequent year increased. These findings support our hypothesis that the polygyny threshold is a dynamic attribute of a population and the cost of polygyny for females varies from year to year.

### The frequency of social polygyny and female survival

The idea that female choice of a paired male depends on a trade-off between benefits and costs is central to the polygamy threshold model and the sexy son hypothesis. Both hypotheses contend that a female is more likely to accept an already mated male when the quality of the breeding situation he provides compensates for the loss of assistance in parental care. Although it received experimental support in a study with pied flycatchers^29^, such a mechanism of choice has been investigated only anecdotally. Moreover, the existing theoretical and empirical (seldom experimental^29^) literature has focused on the variation in benefits, implicitly assuming a fixed cost for the female choice of paired males (but see^13,29^). Thus, the drivers of variation in mate sharing costs have not been studied in this or other species. We posit that such a variation could shape the frequency of social polygyny over time (from 1 year to the next) and space (among different populations). In the study population, we found a positive association between the number of hatchlings that manage to fledge each year and the frequency of social polygyny, suggesting that when rearing conditions are better, more females engage in mating with a polygynous male. Overall, only a few studies have measured the variation in FSP. For example, studies on Sedge Warbler *Acrocephalus schoenobaenus* revealed variation in FSP among different populations (0–19% of polygyny—e.g.^30–32^). In our study population, there was large variation in FSP over the study period (mean = 9.77%, range = 0–27.42%). After years of high FSP values, we found a smaller difference in female survival between monogamous and polygamous females’ and a higher probability of mating in a polygamous state for two consecutive years. The increased survival of polygamous females mainly drove this reduction in the survival difference after years of high FSP (Fig. 2). Therefore, our findings appear to support the FPT hypothesis that the frequency of social polygyny increases when the costs of mate sharing reduce, though experimental evidence is needed. Other hypotheses about the drivers of FSP, such as operational sex ratio^1^ or breeding density^11^, cannot explain our findings because they do not make any link between the FSP and the differential survival of polygamous and monogamous females.

### Probability of local survival by mating status of females

Another piece of evidence in support of the FPT hypothesis comes from the observed lower survival of polygamous females relative to that of monogamous females, which contrasts with that reported in previous works (e.g.,^22,23,33^). This expectation of the polygyny threshold framework, including the FPT hypothesis, is based on the assumption that females pay a cost for mate sharing. Such a cost has been demonstrated in the pied flycatcher and other bird species (e.g.^17,18^), showing that females of polygynous males increase their parental effort throughout the nesting period to compensate for decreased male assistance. In the study population, we know that male help is substantially reduced at secondary nests^28^, and that the body mass of secondary females is on average lower than that of monogamous females (Appendix S3). However, as the estimated parameter is local survival (probability of staying and surviving), we cannot rule out that the difference detected is due to emigration rather than survival. Polygamous females, for example, could be more prone to emigration if they perceive poor breeding conditions due to the male’s lack of assistance. In the polygamous group, we expected secondary females to have a lower likelihood of survival than primary females under the assumption that secondary females need to^18,25^ compensate for possible lower male assistance^34^. However, the possibility that survival secondary females has lower survival than primary females (estimates from model F9) received little support (2.04 ΔAIC_c_ between models F6—same survival of primary and secondary females—and F9 in Table 1). A power analysis of the difference in survival between the primary and secondary females in our study population^14^ suggests that the effect size is likely too small to be detected. Similar to our findings, Lamers et al.^20^ found that primary females have lower annual probabilities of local survival than monogamous females (p_*prim*_ = 0.25 and p_*mon*_ = 0.38, respectively) in a Swedish pied flycatcher population. We also found that, on average, > 1-yo females were less likely to survive from 1 year to the next than 1-yo ones regardless of their mating status. Our population had a considerably higher probability of survival (p_*pol 1-yo*_ = 0.55 and p_*mon 1-yo*_ = 0.63 *versus* p_*pol* >*1-yo*_ = 0.47 and p_*mon* >*1-yo*_ = 0.55) compared to the Swedish population, possibly due to different mortality rates (rather than different site fidelity) as suggested by a study^35^ comparing the dispersal rates from northern and central European populations of pied flycatchers. Contrary to our results, Garamszegi et al.^23^ found that primary and secondary collared flycatcher females had higher local survival probabilities than monogamous females. Most likely, the discrepancy is due to their consideration of the female mating status as a fixed attribute (e.g., assigned as a secondary female if observed at least once in that state) throughout the lifetime. The local survival estimate of the secondary females might thus be inflated because the more years an individual is captured, the higher its probabilities of surviving and being assigned to the group of secondary females. Accordingly, when we analysed our data following the same female categorisation used by Garamszegi et al.^23^, we obtained similar results to theirs (i.e. higher survival of secondary females; data not shown).

### Probability of survival by the mating status of males

If polygynous males invest more effort than monogamous males in reproduction, it would be expected that they pay a cost for polygyny. Contrary to this idea, we found no differences in survival between monogamous and polygynous males, which could be due to several reasons. First, polygynous males simultaneously occupy two (or more) territories (nesting cavities^16^) and may provide food to both primary and secondary females and their broods. However, polygynous pied flycatcher males typically offer little assistance to the secondary brood^16,17,28,36^. Second, if polygynous males have broader home ranges, they might experience higher energy costs and displacement hazards than monogamous males. This scenario does not seem to apply to our study population as primary and secondary females are, in most cases, close neighbours (i.e., < 50 m apart^24,28^). Third, the extra work needed to rear two broods may have carry-over or cascading effects and, as a result, polygynous males might depart and arrive later at the winter quarters and thus be relegated to low-quality habitats^37^. Nevertheless, previous works on the study population indicate that males’ probability of engaging in social polygyny is closely related to an early arrival date^24,26^. Despite opposing views on whether males pay a cost for social polygyny, and despite the fact that our study found no such cost in survival, these costs may be expressed in fitness components other than survival. As an example, Gustafsson et al.^19^ showed an indirect cost for polygynous males in a Swedish population of collared flycatchers by reducing their forehead patch size that made them less attractive to females and less likely to mate the following year.

### Probability of mating status change

We found that the primary and secondary females had the same likelihood of becoming monogamous in the next breeding season. As a result, the probabilities of transitioning from primary to secondary mating status, and vice versa, were the same. The low probability of breeding consecutively in the same mating status (monogamously or polygamously) confirms that mating status is a dynamic individual trait. If it were a fixed characteristic of the individuals, these probabilities would be close to one, regardless of their frequency in the population. The high proportion of monogamous individuals observed in the population reflects the disparity between the chances of breeding in two consecutive years in a monogamous or polygamous status.

### Probability of socially polygamous bond at the first known breeding event

We found that, at the first known breeding event (when captured the first time as an adult breeder in the study population), 1-year-old females had a higher chance of mating with a polygamous male than older ones. This disparity is most likely due to the fact that younger females arrive late, after most males are already paired^11,26^. Another possible explanation is that age-related plumage differences in females signal their experience to males^38^. Similarly, we found that males above 1 year of age were more likely to be polygynous at their first breeding event than younger ones. Most likely, this is because in most pied flycatcher populations 1-yo males, as occur with females, tend to arrive at the breeding areas very late in the season^39–41^. By arriving early, older males have a higher probability of finding better places to nest and/or higher chances of occupying two or more unoccupied nest cavities to become polygynous^24,26,41^, without any role of morphological traits^14^.

Overall, our study provides observational support to the proposed FPT hypothesis revealing a previously undisclosed relationship of the frequency of social polygyny with the quality of rearing conditions, the difference between monogamous and polygamous females’ survival and the probability of between-year changes in mating status in males and females. The theoretical and empirical literature on the polygamy threshold implies that the costs of polygyny for female choice within a population are constant. Our study extends this framework by suggesting the environment as a possible modulator of these costs and, consequently, the polygyny threshold. We call for future research to look into the environmental changes that cause fluctuations in the polygyny threshold and how it varies by individual (males and females) traits.

## Methods

### Study area and study population

Data come from a long-term study of a pied flycatcher population breeding in nestboxes in central Spain (ca. 41°N, 3°W, 1200–1300 m.a.s.l.). The longitudinal data cover the period 1990–2016 (no data for 2003) and include records for 1436 males (yearly mean and SD: 107.4 and 34.2) and 1641 females (yearly mean and SD: 119.7 and 28.6). The study area consists of two plots in two different montane habitats separated by 1.1 km, including 237 nestboxes with an average occupancy rate around 54% (SD = 0.11). One habitat is an old deciduous oak (*Quercus pyrenaica*) forest, and the other one is a managed mixed coniferous (mainly *Pinus sylvestris*) forest. The nestboxes have remained in the same position since 1988 (pinewood) and 1995 (oakwood) (for details, see^42,43^).

### Fieldwork and data collection

Nestboxes were regularly (every 3rd–4th day) checked during the breeding season (from mid-April to the beginning of July) to determine the date of the first egg laid, clutch size, hatching date, and the number of fledglings. Parents were captured with a nestbox trap while incubating (females) or feeding 8-day-old nestlings (both sexes; for details, see^43^ and marked with a numbered metal ring (both sexes). We used a unique combination of colour rings (males only) for individual identification before capture. Many breeding birds (53%) hatched in the nestboxes, and, therefore, their exact age was known^44^. Unringed breeders were aged as first-year or older based on plumage traits following ageing criteria described in^44,]^^45^. All nestlings were ringed at 13 days of age.

Polygamous males were detected when captured and/or individually identified while repeatedly feeding young in two nests (see^24^ for details on capture protocol and mating status classification). We distinguished three classes of females according to their male mating status: (i) monogamous female, i.e. mated with a monogamous male; (ii) primary female, the first mated female of a polygynous male; and (iii) secondary female, the second mated female of a polygynous male. However, in some nests, it was not possible to know with certainty the mating status of the female (14.3% of times) or the male (3.7% of times, see below for how we dealt with this source of uncertainty).

### Ethics declaration

The study was reviewed by the ethical committees at the Doñana Biological Station and the Consejo Superior de Investigaciones Científicas headquarters (Spain) and adhered to Spain standards. All methods were carried out in accordance with relevant guidelines and regulations. Birds were caught and ringed with permission from the Spanish Ministry of Agriculture, Food, Fisheries, and Environment’s Ringing Office. The study complied with (Animal Research: Reporting of In Vivo Experiments) guidelines^46^.

### Multi-event capture-recapture models

We used multi-event capture-recapture (MECR hereafter) models^47^ to test, separately for females and males, how the mating status affected the probability of surviving (and not leaving the area permanently) and the probability of changing, or not, from one mating status to another. The MECR models accommodate uncertainty in state assignment by distinguishing between what is observed (the event) and what is inferred (the state). This approach allows estimating the effects of mating status on the parameters (e.g. probabilities of local survival and change in mating status) while accounting for the uncertainty, as outlined above, due to the unknown mating status of some captured individuals.

MECR models are defined by three types of parameters: Initial State probabilities, Transition probabilities and Event probabilities (details in Appendices S5). As these parameter types may be broken into steps, we considered two Transition steps, Local survival and Mating Status Change, and two Event steps, Recapture and Mating Status Assignment. Accordingly, we considered the following parameters of the MECR model: (i) Initial State, the probability of being in a specific mating status at the first encounter (in our case the first known breeding event of an individual); (ii) Local survival, the probability of surviving and not emigrating permanently from the study area between year *t* and year *t* + 1; (iii) Mating Status Change, the probability that a live bird changes state between year *t* and *t* + 1; (iv) Recapture: the probability of recapture of a live and not permanently emigrated individual; (v) Mating Status Assignment: the probability that the mating status of a captured individual is ascertained in the field (assuming no state misclassification). In this study, we will use the term “parameter” to denote any of the probabilities (see i-v above) estimated in the MECR model. Also, note that, as is often the case, we cannot distinguish the probability of site fidelity from that of surviving. For simplicity, we will often use the term “survival” to refer to “local survival”.

We used the encounter histories of all identified birds breeding in the study area at least once between 1990 and 2016. We ran separate analyses for each sex, considering four biological states for females: live monogamous breeder (MBF), live primary breeder (PBF), live secondary breeder (SBF) and dead or permanently emigrated (†); and five events, numbered as they appear in the encounter histories: (0) non-captured, (1) captured as a monogamous breeder, (2) captured as a primary breeder, (3) captured as a secondary breeder and (4) captured in an unknown mating status. Females of unknown mating status were those for which we did not know the mate’s identity after repeated identification attempts at the nestbox (see details in^24^). These females could be of any mating status, and the mate being absent (e.g. dead after pairing) or very sporadically visiting the nest. For males, however, we considered three biological states: live monogamous breeder (MBM), live polygynous breeder (PBM) and dead or permanently emigrated (†), mediated by four events: (0) non-captured, (1) captured as a monogamous breeder, (2) captured as a polygynous breeder, (3) captured in an unknown mating status. Males of unknown mating status were identified by reading their colour-rings combinations near a nestbox and not captured or seen again during the breeding season. For both sexes, we established two age classes: 1-year-old individuals (1-yo hereafter: 41.74% females; 26.46% males) and individuals older than 1 year (> 1-yo hereafter: 58.26% females; 73.54% males) that we included as a control variable in our capture-recapture models. This classification allowed the inclusion of non-local breeders (immigrants) in our analyses.

Models were built and fitted to the data using E-SURGE 2.2.0^48^. As our data were annually collected and we had no data for 2003, we selected the “Unequal Time Intervals” option to account for the 2002–2004 interval. Details on the probabilistic framework and the limitations of the modelling approach are given in Appendix S4.

### Goodness of fit

Before running the capture-recapture analysis, we preliminary assessed the goodness of fit (GOF) of a general model to the data. Since GOF tests are not available for multi-event models, we tested the GOF of the Cormack-Jolly-Seber (CJS), a model accounting for just two states, alive and dead, and for temporal variation in survival (Transition) and recapture (Event) probabilities, using U-CARE 2.3.2^49^. This approach is conservative because the CJS is coarser than the MECR model. Thus, if the former fits the data well, the latter will fit them. All the GOF tests were run for males and females separately. The global tests were not significant for both males [c^2^ = 72.57, df = 103, p = 0.99; N(0,1) statistic for transient (> 0) =  − 0.49, p = 0.69; N(0,1) signed statistic for trap-dependence = − 0.84, p = 0.99] and females [c^2^ = 76.13, df = 122, p = 0.99; N(0,1) statistic for transient (> 0) = − 2.51, p = 0.69; N(0,1) signed statistic for trap-dependence = − 1.22, p = 0.22], indicating acceptable fits of the Cormack-Jolly-Seber models to the data. For the complete results of 3.SR (transience) and 2.CT (trap-dependence) tests, see Appendix S5.

### Model selection

Model selection was based on Akaike Information Criterion corrected for small sample sizes (AIC_c_)^50^. For each sex, in a preliminary analysis, we built a global model checking that there were no parameter identifiability issues^48^. The structure of the global model was: *Initial State (mating status × time), Local survival (age* + *(mating status × time)), Mating Status Change (age × mating status), Recapture (mating status × time), Mating status Assignment (mating status × time)*.

Our modelling approach consisted of two steps. In *step one*, starting from the global model, we followed a backwards model selection procedure to test various combinations of variables potentially influencing each parameter of the MECR model while simplifying the model’s structure. According to the classic approach for which the recapture part of the model is modelled before that of survival^51,52^, we followed the following order of model selection: Initial State, Mating Status Assignment, Recapture, Mating Status Change, and Local survival. After testing the model structure (set of effects) for a parameter, we set the best structure (lower AIC_c_) for that parameter, and we then tested the models for the following parameter. Thus, at the end of *step one,* we examined the effect of mating status on the biologically relevant parameters, that is, on Local survival and Mating Status Change. In *step two*, we used the simplified model resulting from step one (*final model 1*) to test whether the frequency of the FSP differentially affected the biologically relevant parameters according to the mating status. First, we tested the effects of FSP on Mating Status Change and then on Local survival (by keeping in MSC the same structure of *final model 1*). In the Results section, we reported parameter’ estimates from a model that combined the best final structure (lowest AIC_c_) found on all the parameters, when not stated otherwise.

### Linear regression analysis of FSP and fledging success of hatchlings

We used a GLM model to test whether the FSP depends on the yearly average proportion of hatchlings that fledged. We used the *simulateResiduals* function of the DHARMa^53^ package in R^54^ to confirm the absence of over-dispersion and the good fit of the model.

## Supplementary Information

Supplementary Information.

## Data Availability

The datasets generated during and/or analysed during the current study are available from the corresponding author on reasonable request.
