# Supplementary material for: High frequency of social polygyny reveals little costs for females in a songbird
Source: Sci Rep. 2022 Jan 7;12:277. doi: 10.1038/s41598-021-04423-0 (PMC8742037; doi:10.1038/s41598-021-04423-0)
Supplement: Supplementary file 1 — Supplementary Information. [file 41598_2021_4423_MOESM1_ESM.pdf]

SUPPORTING INFORMATION

Content

Appendix S1. Percentages of polygamous pair-bonds in the study population.......................... 2

APPENDIX S2. Detailed estimates from MECR models 3

Females' estimates.. 3

Males' estimates 6

APPENDIX S3. Probabilistic framework in E-SURGE and limitations of the modelling approach . 10

Females' probabilistic framework... 10

Males' probabilistic framework 13

Limitations of the modelling approach 15

Literature Cited 17

APPENDIX S4. Secondary females have lower body mass than monogamous females 19

APPENDIX S5. Goodness of fit test 21

Females 21

Males.. 23

Table S1... 25

Appendix S1. Percentages of polygamous pair-bonds in the study population

Year	FSP (% of polygamous bonds)
1990	0.057142857
1991	0.056818182
1993	0.074074074
1994	0.096153846
1998	0.072164948
1999	0.066666667
2000	0.068965517
2004	0.091836735
2005	0.274193548
2006	0.25984252
2007	0.213235294
2008	0.132352941
2009	0.19047619
2010	0.082089552
2011	0.117241379
2012	0.086206897
2013	0.151079137
2014	0.127659574
2015	0.026845638

APPENDIX S2. Detailed estimates from MECR models

We report here the complete estimates of probabilities of Initial State, Apparent Survival, Mating Status Change, Recapture, and Mating Status Assignment of females and males. The estimates proceed from a model which combines the best final structure (lowest AIC_c) found on all the parameters, without any effect of the frequency of social polygyny (FSP) (i.e. from *step one* of model selection, see main text). For the model selection of females, this model corresponds to model F6 in Table 1 (see also Table 1 for the notation), whereas for the males it corresponds to model M6. The structure of these models is:

Model F6 *{Initial State (ms x age), Apparent Survival (age + ms (M,P=S)), Mating Status Change (mon.pol1), Recapture (ms + t), Mating status Assignment (ms + t)},*

Model M6 *{Initial State (ms x age), Apparent Survival (i), Mating Status Change (ms), Recapture (ms + t), Mating status Assignment (t)}.*

Females' estimates

Females – Initial State (*age + mating status*). *Est*, Estimate, *CI-*, lower 95% confidence limit, *CI+*, upper 95% confidence limit.

	Est	CI-	CI+
Mon 1yo	0.75	0.72	0.79
Prim 1yo	0.07	0.04	0.14
Sec 1yo	0.17	0.13	0.23
Mon >1yo	0.82	0.79	0.85

Prim > 1yo	0.07	0.04	0.11
Sec > 1yo	0.11	0.08	0.15

Females – Apparent Survival (*age + mating status(Mon, Prim = Sec)*).

	Est	CI-	CI+
Mon 1yo	0.63	0.58	0.68
Pol 1yo	0.55	0.49	0.6
Mon >1yo	0.55	0.52	0.58
Pol >1yo	0.47	0.42	0.51

Females – Mating Status Change. The 95% CIs are shown between the brackets.

	Monogamous	Primary	Secondary
Monogamous	0.71 (0.67 - 0.74)	0.15 (0.13 - 0.17)	0.15 (0.13 - 0.17)
Primary	0.7 (0.63 - 0.75)	0.19 (0.11 - 0.31)	0.11 (0.05 - 0.24)
Secondary	0.7 (0.63 - 0.75)	0.11 (0.05 - 0.24)	0.19 (0.11 - 0.31)

Females – Recapture (*mating status + time*). Some estimates are reported as 0 or 1 for rounding reasons (they are not at the boundary).

		Est	CI-	CI+		Est	CI-	CI+		Est	CI-	CI+
1991	Mon	1	1	1	Prim	0.09	0.01	0.52	Sec	0.09	0.01	0.49
1992	Mon	1	1	1	Prim	0.28	0.08	0.64	Sec	0.27	0.07	0.64
1993	Mon	1	1	1	Prim	0.31	0.09	0.68	Sec	0.3	0.08	0.68
1994	Mon	1	1	1	Prim	0.43	0.16	0.75	Sec	0.42	0.15	0.75
1995	Mon	1	1	1	Prim	0.48	0.16	0.82	Sec	0.47	0.15	0.82
1996	Mon	1	1	1	Prim	0.51	0.15	0.86	Sec	0.51	0.16	0.84
1997	Mon	1	1	1	Prim	0.08	0	0.94	Sec	0.08	0	0.94

1998	Mon	1	1	1	Prim	0.56	0.18	0.88	Sec	0.55	0.19	0.86
1999	Mon	1	1	1	Prim	0.17	0.05	0.47	Sec	0.17	0.05	0.45
2000	Mon	1	1	1	Prim	0.4	0.12	0.76	Sec	0.39	0.13	0.74
2001	Mon	1	1	1	Prim	0.62	0.24	0.89	Sec	0.61	0.24	0.88
2002	Mon	0.75	0.44	0.92	Prim	0	0	0	Sec	0	0	0
2004	Mon	1	1	1	Prim	0.44	0.05	0.92	Sec	0.43	0.07	0.88
2005	Mon	1	1	1	Prim	0.8	0.47	0.95	Sec	0.79	0.48	0.94
2006	Mon	1	1	1	Prim	0.62	0.31	0.86	Sec	0.61	0.31	0.85
2007	Mon	1	1	1	Prim	0.86	0.56	0.97	Sec	0.86	0.53	0.97
2008	Mon	1	1	1	Prim	0.32	0.12	0.62	Sec	0.31	0.11	0.61
2009	Mon	1	1	1	Prim	0.68	0.34	0.9	Sec	0.67	0.4	0.86
2010	Mon	1	1	1	Prim	0.18	0.05	0.48	Sec	0.17	0.04	0.5
2011	Mon	1	1	1	Prim	0.34	0.14	0.62	Sec	0.33	0.12	0.64
2012	Mon	1	1	1	Prim	0.53	0.26	0.79	Sec	0.52	0.28	0.76
2013	Mon	1	1	1	Prim	0.43	0.14	0.78	Sec	0.42	0.18	0.71
2014	Mon	1	1	1	Prim	0.58	0.29	0.82	Sec	0.57	0.24	0.84
2015	Mon	1	1	1	Prim	0.1	0.02	0.39	Sec	0.1	0.02	0.38
2016	Mon	1	1	1	Prim	0.67	0.37	0.87	Sec	0.66	0.31	0.89

Females – Mating Status Assignment (*mating status* + *time*). Some estimate are reported as 0 or 1 for rounding reasons (they are not at the boundary).

		Est	CI-	CI+		Est	CI-	CI+		Est	CI-	CI+
1991	Mon	1	0.99	1	Prim	0.88	0.4	0.99	Sec	0.76	0.26	0.97
1992	Mon	1	0.98	1	Prim	0.69	0.31	0.92	Sec	0.49	0.22	0.77
1993	Mon	0.99	0.95	1	Prim	0.45	0.17	0.77	Sec	0.26	0.11	0.51
1994	Mon	0.99	0.94	1	Prim	0.42	0.16	0.74	Sec	0.24	0.1	0.47
1995	Mon	1	0.97	1	Prim	0.63	0.3	0.88	Sec	0.43	0.2	0.69
1996	Mon	0.61	0.48	0.73	Prim	0.01	0	0.08	Sec	0.01	0	0.03
1997	Mon	0.71	0.58	0.81	Prim	0.02	0	0.11	Sec	0.01	0	0.04
1998	Mon	0.99	0.94	1	Prim	0.43	0.17	0.74	Sec	0.25	0.1	0.49
1999	Mon	0.99	0.93	1	Prim	0.39	0.12	0.74	Sec	0.21	0.07	0.48
2000	Mon	0.99	0.95	1	Prim	0.46	0.18	0.78	Sec	0.27	0.11	0.53

2001	Mon	0.87	0.71	0.95	Prim	0.05	0.01	0.25	Sec	0.02	0	0.11
2002	Mon	0.09	0.03	0.25	Prim	0	0	0.01	Sec	0	0	0
2004	Mon	0.99	0.97	1	Prim	0.59	0.26	0.85	Sec	0.38	0.18	0.63
2005	Mon	1	0.99	1	Prim	0.77	0.49	0.92	Sec	0.59	0.39	0.77
2006	Mon	1	0.98	1	Prim	0.71	0.42	0.9	Sec	0.52	0.33	0.7
2007	Mon	1	0.99	1	Prim	0.75	0.47	0.91	Sec	0.56	0.37	0.73
2008	Mon	0.99	0.96	1	Prim	0.49	0.2	0.79	Sec	0.29	0.14	0.52
2009	Mon	1	0.98	1	Prim	0.63	0.34	0.85	Sec	0.42	0.24	0.62
2010	Mon	0.99	0.96	1	Prim	0.47	0.2	0.76	Sec	0.27	0.12	0.52
2011	Mon	0.99	0.96	1	Prim	0.5	0.21	0.79	Sec	0.3	0.14	0.54
2012	Mon	0.99	0.96	1	Prim	0.48	0.22	0.76	Sec	0.29	0.14	0.51
2013	Mon	1	0.98	1	Prim	0.65	0.34	0.87	Sec	0.44	0.24	0.67
2014	Mon	0.99	0.97	1	Prim	0.57	0.28	0.82	Sec	0.36	0.2	0.56
2015	Mon	0.99	0.96	1	Prim	0.48	0.18	0.8	Sec	0.28	0.11	0.55
2016	Mon	1	0.98	1	Prim	0.62	0.34	0.84	Sec	0.42	0.25	0.61

Males' estimates

Males – Initial State (*age x mating status*). *Est*, Estimate, *CI-*, lower 95% confidence limit, *CI+*, upper 95% confidence limit.

	Est	CI-	CI+
Mon 1yo	0.99	0.97	1
Pol 1yo	0.01	0	0.03
Mon >1yo	0.96	0.95	0.97
Pol > 1yo	0.04	0.03	0.05

Males – Apparent Survival (*constant*).

	Est	CI-	CI+
Mon and Pol	0.54	0.52	0.56

Males – Mating Status Change (*mating status*).

	Est	CI-	CI+
Mon to Mon	0.79	0.75	0.83
Pol to Mon	0.85	0.74	0.92
Pol to Pol	0.15	0.08	0.26
Mon to Pol	0.21	0.17	0.25

Males – Recapture (*mating status* + *time*).

		Est	CI-	CI+		Est	CI-	CI+
1991	Mon	0.98	0.82	1	Pol	0.28	0.04	0.77
1992	Mon	0.98	0.84	1	Pol	0.28	0.09	0.61
1993	Mon	0.98	0.83	1	Pol	0.26	0.08	0.57
1994	Mon	0.97	0.78	1	Pol	0.17	0.05	0.45
1995	Mon	0.96	0.68	1	Pol	0.16	0.04	0.49
1996	Mon	0.47	0.31	0.63	Pol	0.01	0	0.04
1997	Mon	0.72	0.49	0.87	Pol	0.02	0	0.12
1998	Mon	0.92	0.66	0.99	Pol	0.08	0.02	0.29
1999	Mon	0.75	0.55	0.88	Pol	0.02	0	0.13
2000	Mon	0.98	0.86	1	Pol	0.28	0.07	0.65
2001	Mon	0.82	0.51	0.95	Pol	0.03	0	0.22
2002	Mon	0.03	0	0.19	Pol	0	0	0
2004	Mon	0	0	0	Pol	0	0	0
2005	Mon	0.99	0.95	1	Pol	0.56	0.3	0.79
2006	Mon	1	0.96	1	Pol	0.62	0.31	0.86

2007	Mon	0.99	0.93	1	Pol	0.46	0.23	0.71
2008	Mon	0.99	0.89	1	Pol	0.32	0.12	0.62
2009	Mon	0.99	0.89	1	Pol	0.36	0.12	0.7
2010	Mon	0.98	0.85	1	Pol	0.24	0.08	0.53
2011	Mon	0.95	0.7	0.99	Pol	0.12	0.03	0.35
2012	Mon	0.96	0.73	1	Pol	0.16	0.05	0.4
2013	Mon	0.99	0.91	1	Pol	0.42	0.16	0.73
2014	Mon	0.99	0.94	1	Pol	0.54	0.21	0.84
2015	Mon	0.98	0.9	1	Pol	0.31	0.08	0.69
2016	Mon	0.99	0.9	1	Pol	0.43	0.15	0.76

Males – Mating Status Assignment (*mating status* + *time*). Some estimate are (1) are estimated at the boundary (indicating a very high probability of mating status assignment).

		Est	CI-	CI+		Est	CI-	CI+
1991	Mon	0.95	0.86	0.98	Pol	0.95	0.86	0.98
1992	Mon	1	1	1	Pol	1	1	1
1993	Mon	0.99	0.93	1	Pol	0.99	0.93	1
1994	Mon	1	1	1	Pol	1	1	1
1995	Mon	1	1	1	Pol	1	1	1
1996	Mon	0.95	0.88	0.98	Pol	0.95	0.88	0.98
1997	Mon	0.98	0.86	1	Pol	0.98	0.86	1
1998	Mon	0.61	0.51	0.7	Pol	0.61	0.51	0.7
1999	Mon	0.99	0.93	1	Pol	0.99	0.93	1
2000	Mon	1	1	1	Pol	1	1	1
2001	Mon	0.99	0.92	1	Pol	0.99	0.92	1
2002	Mon	0.98	0.9	1	Pol	0.98	0.9	1
2004	Mon	0.75	0.24	0.97	Pol	0.75	0.24	0.97
2005	Mon	0.96	0.89	0.98	Pol	0.96	0.89	0.98
2006	Mon	0.98	0.93	1	Pol	0.98	0.93	1
2007	Mon	0.95	0.9	0.98	Pol	0.95	0.9	0.98
2008	Mon	0.97	0.92	0.99	Pol	0.97	0.92	0.99

2009	Mon	0.98	0.93	0.99	Pol	0.98	0.93	0.99
2010	Mon	0.98	0.94	1	Pol	0.98	0.94	1
2011	Mon	0.98	0.93	0.99	Pol	0.98	0.93	0.99
2012	Mon	0.99	0.94	1	Pol	0.99	0.94	1
2013	Mon	0.98	0.93	1	Pol	0.98	0.93	1
2014	Mon	0.98	0.93	0.99	Pol	0.98	0.93	0.99
2015	Mon	0.98	0.93	0.99	Pol	0.98	0.93	0.99
2016	Mon	0.98	0.94	0.99	Pol	0.98	0.94	0.99

APPENDIX S3. Probabilistic framework in E-SURGE and limitations of the modelling approach

In E-SURGE, after loading the data and setting a few options within the program (e.g. number of states, number of events, number of age classes, time interval length, covariate file), the procedure to run a model consists of two steps. First, using the GEPAT (GEnerator of PATtern of elementary matrices;[1]) the user needs to set up the probability matrices. Note that all the matrices are row-stochastic meaning that each row satisfies the condition of a discrete probability distribution (their sum being equal to one). Then, in each row, the user needs to choose which parameter is redundant (i.e. is complementary to the sum of the others), and the setting of a probability matrix can be modified. As an example, we have used an alternative configuration of the Mating Status Change matrix to run models where we tested the relationship between the probability of remaining in a given state and the frequency of social polygyny (e.g. models F-FSP1 and M-FSP1 in Table 2). Second, using the GEMACO (GEnerator of Matrices of COnstraints; [1]), the user defines the set of effects on each parameter.

Females' probabilistic framework

We used five probability matrices to model the apparent survival and mating status transition of females, and to relate female observations to the true underlying state: (i) Initial State, (ii) Apparent Survival, (iii) Mating Status Change, (iv) Recapture, and (v) Mating Status Assignment. At a given time, a female can be in one of the mutually exclusive four biological states: MBF= monogamous breeder female, PBF= primary breeder female, SBF= secondary

breeder female, †= Dead; and five events: (0) non-captured, (1) captured as monogamous breeder, (2) captured as primary breeder, (3) captured as secondary breeder and (4) captured in an unknown mating status. The Initial State matrix defines the probabilities of being in one of the three biological states (excluding the dead state) at the first capture. The Apparent Survival matrix defines the probabilities, specific to each biological state, of surviving from one session to the next (t -> t+1), and the Mating Status Change matrix defines the probabilities of changing mating status. Although we interpret the apparent survival and mating status change as two probabilities of t-> t + 1 interval, they are separated in time within the probability matrices. We assume that an individual in a certain state has a certain probability of surviving the interval and, as it survives, of transitioning to any other state just before time t+1 (see "q" in the matrix notation to indicate a short period). Then, conditioned on being alive at time t+1, an individual has a state-specific probability of being detected (Recapture matrix) and, conditioned on being detected, a state-specific probability of being assessed in one of the four possible events (excluding 0, Mating Status Assignment).

In the notation of the matrices, the Greek symbols (e.g. π) represent the probabilities that are directly estimated by the MECR model and their complementary probabilities (e.g. 1-π, indirectly estimated).

	t	MBF	PBF	SBF
Initial State =		π	π	1−π

Apparent Survival =

t \ t+1-q	MBF	PBF	SBF	†
MBF	ϕ	0	0	$1-\phi$
PBF	0	ϕ	0	$1-\phi$
SBF	0	0	ϕ	$1-\phi$
†	0	0	0	1

Mating Status Change =

t+1-q \ t+1	MBF	PBF	SBF	†
MBF	$1-\psi$	ψ	ψ	0
PBF	ψ	$1-\psi$	ψ	0
SBF	ψ	ψ	$1-\psi$	0
†	0	0	0	1

Recapture =

t+1 \ t+1	0	MBF	PBF	SBF
MBF	$1-\delta$	δ	0	0
PBF	$1-\delta$	0	δ	0
SBF	$1-\delta$	0	0	δ
†	1	0	0	0

Mating Status Assignment =

t+1 \ t+1	0	1	2	3	4
0	1	0	0	0	0
MBF	0	γ	0	0	$1-\gamma$
PBF	0	0	γ	0	$1-\gamma$
SBF	0	0	0	γ	$1-\gamma$

Males' probabilistic framework

We used five probability matrices to model the apparent survival and mating status transition of males, and to relate male observations to the true underlying state: (i) Initial State, (ii) Apparent Survival, (iii) Mating Status Change, (iv) Recapture, and (v) Mating Status Assignment. At a given time, a male can be in one of the mutually exclusive three biological states: MBM= monogamous breeder male, PBM= polygynous breeder male, †= Dead; and four events: (0) non-captured, (1) captured as monogamous breeder, (2) captured as polygynous breeder, (3) captured in an unknown mating status. For details on the probability matrices see the previous section.

Initial State =

t	MBM	PBM
	π	$1-\pi$

Apparent Survival =

t \ t+1-q	MBM	PBM	†
MBM	ϕ	0	$1-\phi$
PBM	0	ϕ	$1-\phi$
†	0	0	1

Mating Status Change =

t+1-q \ t+1	MBM	PBM	†
MBM	$1-\psi$	ψ	0
PBM	ψ	$1-\psi$	0
†	0	0	1

Recapture =

t+1 \ t+1	0	MBM	PBM
MBM	$1-\delta$	δ	0
PBM	$1-\delta$	0	δ
†	1	0	0

Mating Status Assignment =

t+1 \ t+1	0	1	2	3
0	1	0	0	0
MBM	0	γ	0	$1-\gamma$
PBM	0	0	γ	$1-\gamma$

Limitations of the modelling approach

First, a hypothesis we were unable to test is that polygyny determines future reproduction, i.e. the likelihood of breeding in the coming years. In a previous analysis, we tried to test this hypothesis about polygyny's cost to future reproduction using a multi-state capture-recapture framework with a non-observable state [2–5] for individuals that skipped a breeding season. Unfortunately, due to problems with parameter identifiability, we had to abandon the idea. Future reproduction is yet another important component of fitness, and it is worth evaluating the hypothesis that it is affected by polygyny. We recommend that future studies include data on both the mating status and tagged animal movement.

Second, arrival time, and concomitantly [6], breeding date, are related to breeding success and female social mating status in *Ficedula* flycatchers [7–9]. Thus, laying date may exert a confounding effect on the survival probability of individuals of different mating status. To properly account for this effect, we should have considered laying date as a time-varying individual covariate. However, this kind of effects is not yet handled by frequentist capture-recapture models because of missing data for each one of the non-detection events of marked individuals [10]. One possible alternative could have been to calculate the average of individual laying dates and test its effect on survival [9]. However, we discarded this option because both

arrival time and mating status are dynamic, not fixed, characteristics of the individuals [8,11]. Furthermore, arrival and laying dates are rarely, if ever, repeatable in the species [8,12,13]. Despite this shortcoming, we feel confident that our findings do suggest a survival cost for females contingent on their mating status, regardless of any associated effect of breeding date on this parameter. Substantial support in this direction comes from the observation that survival costs to primary and secondary females are similar (cf. model s1and s3 in Table S1), even though primary females typically breed earlier in the season compared to secondary females [7,14,15].

Third, our modelling approach does not allow for mating status misclassification and this might be a concern if, for example, polygynous males are wrongly classified as monogamous or vice versa. However, we assume this is negligible in our study system as we have focused on social polygamy (regardless of genetic paternity), which we have exhaustively monitored in the field. Individuals of unknown mating status are those for which the pair-bonding status has not been unequivocally ascertained despite the exhaustive effort in the field. This may occur by different reasons (e.g. transiency, mortality or emigration within the breeding season) that make some males less susceptible to being captured than others. If our reasoning is correct, we should expect that the probability of mating status assignment decreases from monogamous to primary and secondary females as the time a male spends attending its social mate decreases in this order. This expectation is fully confirmed by our estimates of State Assignment probabilities (see Appendix S2).

Finally, our analyses have been performed using two age classes to not exclude immigrant adults and to maximize the sample size. We cannot discard that age has a more intricate effect on the probability of being polygynous, as well as on the interplay between mating status and survival and breeding propensity. Future studies are needed to clarify this specific aspect.

Literature Cited

1. Choquet, R. Automatic generation of multistate capture recapture models. *The Canadian Journal of Statistics* **36**, 43–57 (2008).

2. Kendall, W. L. & Nichols, J. D. Estimating State-Transition Probabilities for Unobservable States Using Capture- Recapture/Resighting Data. *Ecology* **83**, 3276–3284 (2002).

3. Fujiwara, M. & Caswell, H. A general approach to temporary emigration in mark-recapture analysis. *Ecology* **83**, 3266–3275 (2002).

4. Schaub, M., Gimenez, O., Schmidt, B. R. & Pradel, R. Estimating Survival and Temporary Emigration in the Multistate Capture-Recapture Framework. *Ecology* **85**, 2107–2113 (2004).

5. Sanz-Aguilar, A. *et al.* Studying the reproductive skipping behavior in long-lived birds by adding nest inspection to individual-based data. *Ecological Applications* **21**, 555–564 (2011).

6. Potti, J. & Montalvo, S. Male arrival and female mate choice in pied flycatchers (Ficedula hypoleuca) in central Spain. *Ornis Scandinavica* **22**, 45–54 (1991).

7. Lundberg, A. & Alatalo, R. V. *The pied flycatcher*. (T & A D Poyser, 1992).

8. Potti, J. Arrival time from spring migration in male pied flycatchers: individual consistency and familial resemblance. *The Condor* **100**, 702–708 (1998).

9. Garamszegi, L. Z., Torok, J., Michl, G. & Moller, A. Female survival, lifetime reproductive success and mating status in a passerine bird. *Oecologia* **138**, 48–56 (2004).

10. Gimenez, O. *et al.* Nonparametric estimation of natural selection on a quantitative trait using mark recapture data. *Evolution* **60**, 460–466 (2006).

11. Moreno, J., Veiga, J. P., Romasanta, M. & Sanchez, S. Effects of maternal quality and mating status on female reproductive success in the polygynous spotless starling. *Animal Behaviour* **64**, 197–206 (2002).

12. Montalvo, S. & Potti, J. Breeding Dispersal in Spanish Pied Flycatchers Ficedula hypoleuca. *Ornis Scandinavica* **23**, 491–498 (1992).

13. Both, C., Burger, C., Ouwehand, J., Samplonius, J. M. & Bijlsma, R. G. Delayed age at first breeding and experimental removals show large non-breeding surplus in pied flycatchers. *Ardea* **105**, 43–61 (2017).

14. Canal, D., Jovani, R. & Potti, J. Male decisions or female accessibility? Spatiotemporal patterns of extra pair paternity in a songbird. *Behavioral Ecology* **23**, 1146–1153 (2012).

15. Canal, D., Schlicht, L., Manzano, J., Camacho, C. & Potti, J. Socio-ecological factors shape the opportunity for polygyny in a migratory songbird. *Behavioral Ecology* **31**, 598–609 (2020).

APPENDIX S4. Secondary females have lower body mass than monogamous females

We used a Gaussian GLM in R to test for difference between the body mass of incubating secondary and monogamous females during the study period. We included as covariables the age (1yo or >1yo) and the laying date. We checked the good fit and assumption of the model using the function simulateResiduals() from the package DHARMa (see main text for details and references).

We found a significantly lower body mass for secondary females relative to monogamous females (Fig. S1).

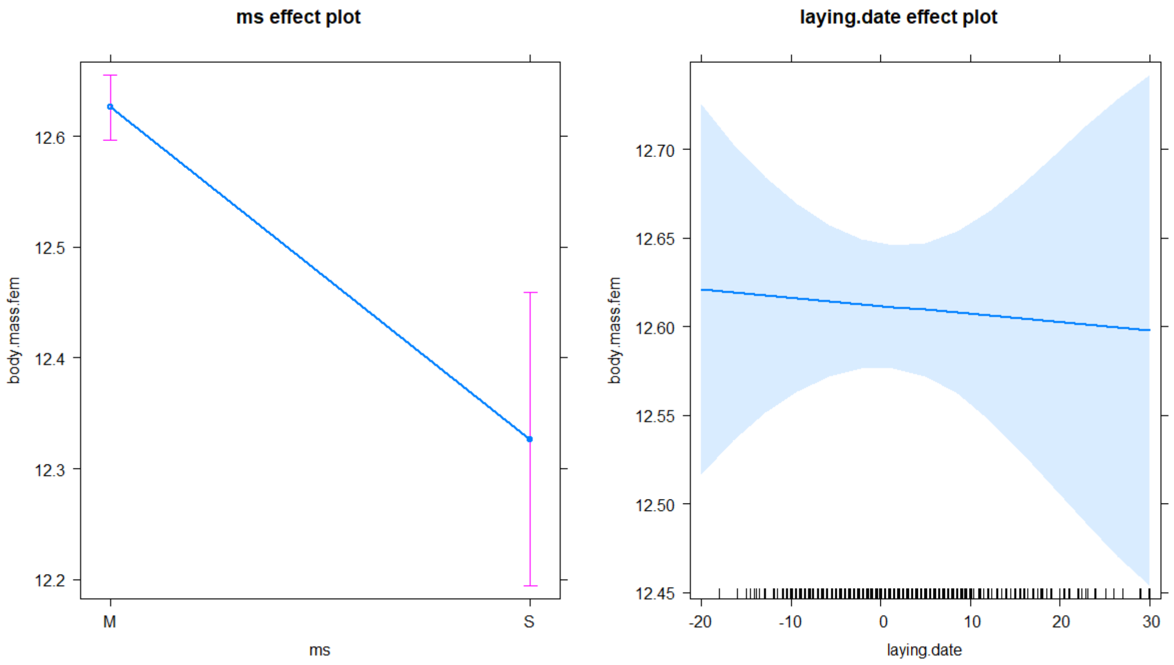

Figure S1. Partial effects of the predictors of the model testing the differential body mass of monogamous and secondary females. Notation: *body.mass.fem*, body mass of females during the incubation, *ms*, mating status, *M*, monogamous females, *S*, secondary females, *laying.date*, laying date.

The complete summary of the model is:

> summary(glm1)

Call:

glm(formula = body.mass.fem ~ ms + fsp + laying.date, data = dati)

```
Deviance Residuals:
     Min        1Q    Median        3Q       Max
-1.88601  -0.42898  -0.04788   0.39801   2.65955

Coefficients:
              Estimate Std. Error t value Pr(>|t|)
(Intercept) 12.7132099  0.0273271 465.224  < 2e-16 ***
msS         -0.2586728  0.0698284  -3.704 0.000218 ***
fsp         -0.7463737  0.1967504  -3.794 0.000153 ***
laying.date -0.0004961  0.0024555  -0.202 0.839901
---
Signif. codes:  0 ‘***’ 0.001 ‘**’ 0.01 ‘*’ 0.05 ‘.’ 0.1 ‘ ’ 1

(Dispersion parameter for gaussian family taken to be 0.3921161)

    Null deviance: 743.19  on 1864  degrees of freedom
Residual deviance: 729.73  on 1861  degrees of freedom
  (557 observations deleted due to missingness)
AIC: 3552.6

Number of Fisher Scoring iterations: 2
```

APPENDIX S5. Goodness of fit test

Females

Global TEST, number of groups =2 (Captured as one-year old birds, captured as older than a year birds)

df =122

Quadratic Chi2 =76.1319

->P-level=0.99963

N(0,1) statistic for transient(>0) =-2.5057

->P-level, two-sided test =0.012219

->P-level, one-sided test for transience =0.99389

N(0,1) signed statistic for trap-dependence =-1.2242

->P-level, two-sided test =0.22087

TEST3.SR, group 1 = Captured as one-year old birds

N(0,1) statistic for transient(>0) =-1.4698

P-level, two-sided test =0.1416

P-level, one-sided test for transience =0.9292

****

TEST3.SR, group 2 = captured as older than a year birds

N(0,1) statistic for transient(>0) =-2.0738

P-level, two-sided test =0.038096

P-level, one-sided test for transience =0.98095

****

TEST2.CT, group 1 = Captured as one-year old birds

N(0,1) signed statistic for trap-dependence =-0.67792

trap-happiness<0 trap-shyness>0

P-level, two-sided test =0.49782

****

TEST2.CT, group 2 = captured as older than a year birds

N(0,1) signed statistic for trap-dependence =-1.0719

trap-happiness<0 trap-shyness>0

P-level, two-sided test =0.28377

Males

Global TEST, number of groups =2 (Captured as one-year old birds, captured as older than a year birds)

df =103

Quadratic Chi2 =72.5682

->P-level=0.99002

N(0,1) statistic for transient(>0) =-0.48922

->P-level, two-sided test =0.62469

->P-level, one-sided test for transience =0.68766

N(0,1) signed statistic for trap-dependence =-0.84183

->P-level, two-sided test =0.39988

TEST3.SR, group 1 = Captured as one-year old birds

N(0,1) statistic for transient(>0) =-0.3316

P-level, two-sided test =0.74019

P-level, one-sided test for transience =0.62991

****

TEST3.SR, group 2 = captured as older than a year birds

N(0,1) statistic for transient(>0) =-0.35998

P-level, two-sided test =0.71886

P-level, one-sided test for transience =0.64057

****

TEST2.CT, group 1 = Captured as one-year old birds

N(0,1) signed statistic for trap-dependence =1.1322

trap-happiness<0 trap-shyness>0

P-level, two-sided test =0.25756

****

TEST2.CT, group 2 = captured as older than a year birds

N(0,1) signed statistic for trap-dependence =-1.7665

trap-happiness<0 trap-shyness>0 P-level, two-sided test =0.077305

Table S1

TABLE S1. Model selection for Initial State, Mating Status Assignment, and Recapture probabilities. For each model, we report the deviance, the number of estimable parameters (*np*), the Akaike Information Criterion corrected for small sample sizes (AIC_c) and the difference in AIC_c between the current model and best model with the lowest AIC_c of the current parameter (ΔAIC_c). Note that not all the nested models have been run because: (i) some of them were illogical (e.g. the frequency of monogamous and polygamous individuals differs considerably for which reason the Initial State always considered a mating status effect), and (ii) some effect was logical but could not be entered in the global model for parameter identifiability issues (e.g. see the "age" effect in FIS1 that is not present in the most parameterized model).

**Females – Initial State**						**Males – Initial State**					
no.	Model	np	Dev	AIC_c	ΔAIC_c	no.	model	np	Dev	AIC_c	ΔAIC_c
FIS1	*age x ms*	245	8652.95	9185.31	0	MIS1	*age*	155	5546.79	5876.61	0
FIS2	*Ms*	243	8660.35	9188	2.68	MIS2	*ms*	154	5556.82	5884.38	7.77
FIS3	*ms x t*	293	8608.37	9255.97	70.66	MIS3	*ms x t*	177	5518.63	5898.69	22.07
**Females – Mating Status Assignment**						**Males – Mating Status Assignment**					
FMSA1	*ms + t*	195	8699.25	9115.66	0	MMSA1	*t*	131	5559.46	5835.5	0
FMSA2	*T*	193	8746.66	9158.51	42.85	MMSA2	*ms + t*	132	5558.92	5837.17	1.68
FIS3	*ms x t*	245	8652.95	9185.31	69.66	MIS3	*ms x t*	155	5546.79	5876.61	41.12
FMSA3	*Ms*	170	8918.13	9278.04	162.38	MMSA3	*ms*	108	5743.71	5969.18	133.68
FMSA4	*I*	168	9050.22	9405.65	289.99	MMSA4	*i*	107	5747.26	5970.55	135.05
**Females – Recapture**						**Males – Recapture**					
FR1	*ms + t*	147	8742.87	9051.65	0	MR1	*ms + t*	109	5580.68	5808.32	0
FR2	*Ms*	123	8818.93	9075.21	23.56	MR2	*t*	108	5605.75	5831.21	22.89
FR3	*I*	121	8841.77	9093.71	42.06	MR3	*ms*	85	5657.64	5833.47	25.14
FR4	*T*	145	8801.1	9105.48	53.82	MMSA1	*ms x t*	131	5559.46	5835.5	27.17
FMSA1	*ms x t*	195	8699.25	9115.66	64	MR4	*i*	84	5700.68	5874.37	66.04

Model notation: +, additive relationship, *x*, non-additive relationship, *ms*, different prob of change from one mating status to another or a different survival prob for each mating status, *t*, time; *i*, no effect, *age*, two-classes age effect (1-yo; >1-yo).
